# Measurement of single Coffea canephora leaf spectro-polarimetric bidirectional reflectance factor dataset

**DOI:** 10.1016/j.dib.2024.110204

**Published:** 2024-02-20

**Authors:** Begzsuren Tumendemberel, Luvsanbat Khurelbaatar, Yukihiro Takahashi, Turtogtokh Tumenjargal, Erdenebaatar Dashdondog

**Affiliations:** a Department of Physics, National University of Mongolia, Ulaanbaatar, Mongolia; b Department of Cosmosciences, Graduate school of Science, Hokkaido University, Sapporo, Japan; c Space Science Center, Institue of Astronomy and Geophysics, MAS, Ulaanbaatar, Mongolia

**Keywords:** Single leaf BRDF, Spectral imaging, Polarization, Goniometer

## Abstract

This work aims to connect polarization techniques with directional relations of the leaf Bidirectional Reflectance Distribution Function (BRDF) by differentiating specular and diffuse reflectance. To do this, a single leave BRDF of Coffea canephora Pierre (Coffee) was captured by a Liquid Crystal Tuneable Filter (LCTF) camera in the 460-780 nm wavelength range with a linear polarizer. The advantage of using an image of the multispectral LCTF camera is that it is able to crop surface area of a leaf, which means it can select an arbitrary size of the field of view and identify the leaf area. We have been building the automatic goniometer with LCTF camera in a laboratory for complete BRDF measurement.

Specifications Table

**Table:** 

Subject	Agriculture engineering Analytical Chemistry: Spectroscopy Optics
Specific subject area	A leaf BRDF measurement
Data format	Analyzed plot
Type of data	Table, Chart, Graph
Data collection	Data is collected at Hokkaido University, using LCTF camera, automatic goniometer, coffee leaf from Hokkaido, Spring 2017
Data source location	Department of Cosmosciences, Graduate School of Science, Hokkaido University, Kita 10-Nishi 8, Kita-Ku, Sapporo, Hokkaido ZIP: 060-0810
Data accessibility	Repository name: Mendeley data Data identification number: 10.17632/nv8rxdbw7k.2 Direct URL to data: https://data.mendeley.com/datasets/nv8rxdbw7k/2

## 1 Value of the Data

• The LCTF with built-in automatic goniometer was camera used for the first time in BRDF measurements. It is also the first time a multi-channel camera has been applied to leaf BRDF measurements, and it is likely to be a benchmark for other researchers.
• Vegetation BRDF measurements are fundamental for agricultural remote sensing from space and drone imaging systems, and depending on the angle of reflection, some pixels may shine. Showing that the specular reflection on the leaf surface can be distinguished with the polarization technique makes this article worthwhile.
• For the first time, we measured both complete leaf BRDF intensity and polarization of reflecting light. Unpolarized BRF were stable, so polarization techniques allow us to make a few measurements to estimate a leaf's biochemical contents.

## 2 Background

The first complete leaf Bidirectional Reflectance Distribution Function (BRDF) measurements were made by [1] and used incident radiation of painted barium sulfate (BaSO4) plates to calculate relative reflectance. Long-distance measurements are often collected from satellites, planes, or drones and must consider atmospheric radiative transfer. Medium-distance measurements often focus on individual plants to characterize leaf area index and leaf angle distribution. Short-distance or single leaf is usually measured in a laboratory. Short-distance measurement is fundamental for agricultural remote sensing. We considered the leaf optical reflection model, polarization property, and a method to determine leaves’ surface roughness, which usually measured using the scanning electron microscope (SEM) by a digital processing technique.

## 3 Data Description

**Fig. 1:**
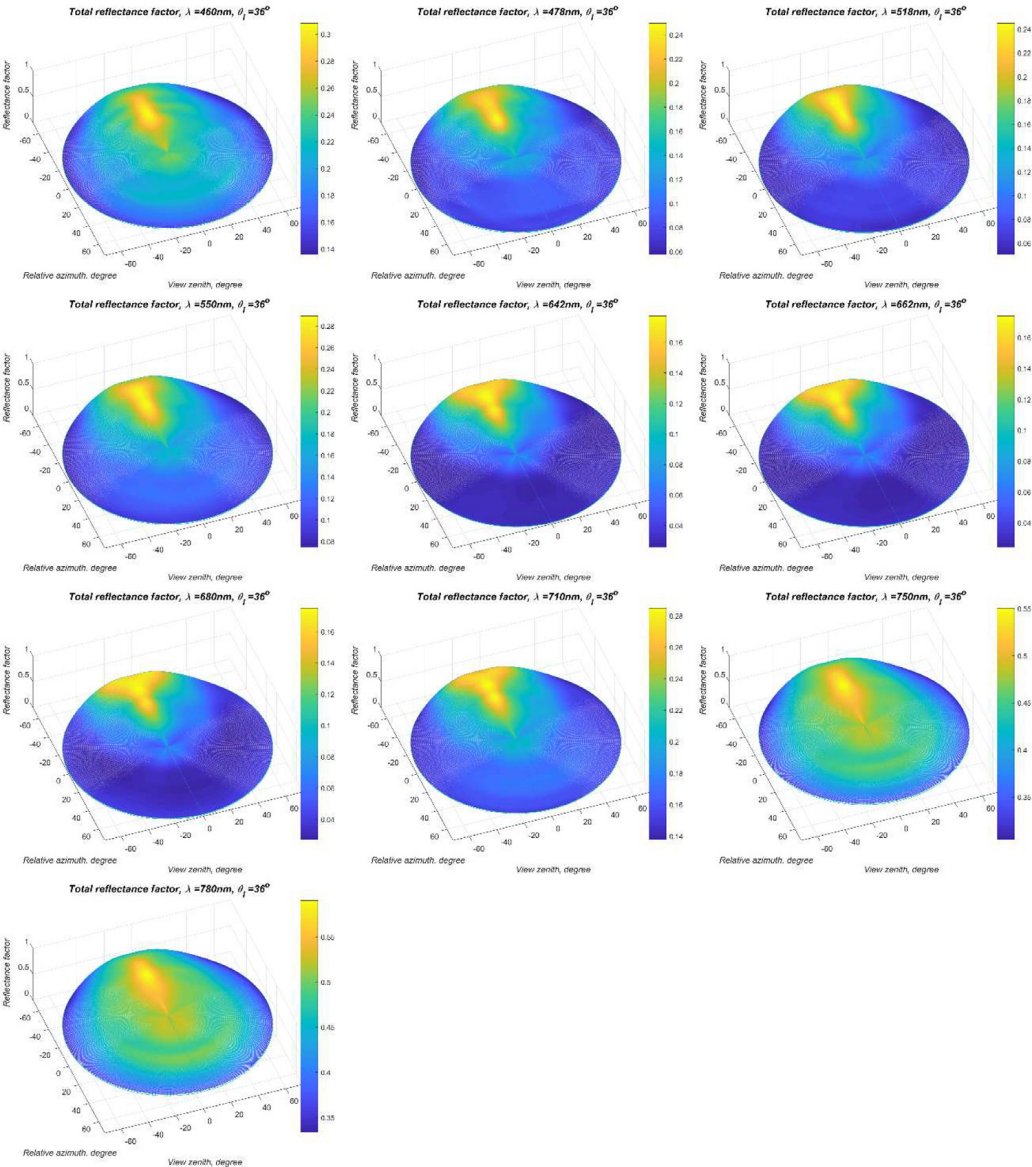
Polar plots of measured the total (the sum of polarized and unpolarized reflectance factors) BRF values of (top) a $\theta_{i}=36^{o}$ at wavelength 480, 478, 518, 550, 642, 662, 680, 710, 750, 780 nm.

We chose a smaller area (5x5 pixels) for coffee because the surface was curved convex at a macro level. The selected section area adjusts the number of pixels as constant as possible. The coffee leaves look shiny because of the light reflection of its wax layer. A total of nine coffee leaves were measured for full BRDF measurements with LCTF camera. The reflectance factor from illumination angle, reflection angle, wavelength (460-780nm), polarization (0^o^, 45^o^, 90^o^, 135^o^), and zenith angle (0^o^, 12^o^, 24^o^, 36^o^, 48^o^, 60^o^, 72^o^). Measurement results of the average of nine coffee leaf total reflectance factor for $\theta_{i}=24^{o}$ is show in Fig. 1.

## 4 Experimental Design, Materials and Methods

### 4.1 Data acquisition

We used the spaceborne multispectral camera with Liquid Crystal Tunable Filter (LCTF: Genesia Corporation) to measure the reflectance of a single leaf in a laboratory. An LCTF camera is installed on the first Earth-observation microsatellite of the Philippines (DIWATA-1) and the Rising-2 microsatellite [2]. This camera works on wavelength bands from 460 nm to 780 nm with a sampling interval of 1nm. The pixel size of the CCD element is the spatial resolution of 656 x 494 pixels. The horizontal and vertical viewing angles are 68.78^o^, and 50.34^o^ degrees, respectively. LCTF camera has two output files: 8 bit of bitmap image and 10 bits of tabular data with comma-separated values (CSV).

Digital numbers of camera pixels were calibrated with the spectral radiance L (W/m2/sr/nm) in such a method as to be able to hold it as an accurate Spectro-radiometric measurement in a simple application. The calibration used the integrating sphere (Labsphere) with a halogen HES -150 lamp, that is a simple, yet often undefined instrument for measuring optical band radiation. The function of an integrating sphere is to integrate radiant flux spatially. The radiance distribution of integrating sphere output can adjust a constant, and the brightness values at each wave frequency are linearly proportional to total intensity. The detailed steps of the calibration method is described in [3] and our camera shows reliable results and the pixel response of CCD cameras linearly when compared with measurements taken simultaneously with other spectroradiometers.

The radiance of one pixel is defined by how many light photons can be harvested by CCD element over the complete band targeted. It can be found from the experimental calibration as:

(1) $$L\left(\lambda ,\alpha \right)=\frac{C_{\left(G,\;\lambda \right)}}{\tau_{e}}\cdot \left(DN\left(\lambda ,\alpha \right)-B\right)\cdot \frac{1}{2T_{\left(\lambda \right)}}\cdot A_{adj}\left(\alpha \right)$$

**Fig. 2:**
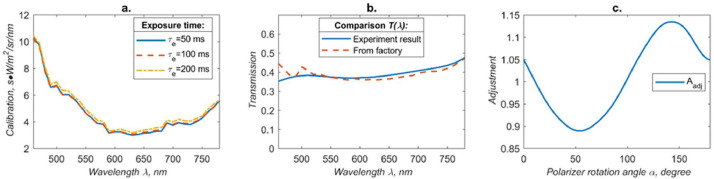
a. The calibration coefficient for radiance measurement by LCTF camera at gain 256. b. $T_{\left(\lambda \right)}$and comparison between factory's specification and laboratory experimental result. c. Polarization adjustment coefficient which is caused by tunable filter.

Eq. (1) where DN(λ)digital number of the pixel, B is background, τ_e_ is exposure time, C _(G, λ)_ is the calibration coefficient, T_(λ)_ is a transmission coefficient of linear polarizing film, A_adj_ (α) is the adjustment coefficient of rotating polarizer axis. The result of the calibration results is plotted in Fig. 2. Motorized linear polarizer was putted in front of LCTF camera lens, normally when making a calibration and doing the measurement at every different angles of rotation.

### 4.2 The automatic goniometer

The optical property of the leaf is usually studied using short distance or small target goniometers (for instance: [4,5] and measured in a laboratory. Darkroom measurements supply much better control of the light source incidence angle than outside field measurements, and also it can be more precisely defined the diffuse compounds [6]. There are thousands of combinations of incident light and observation direction, due to this reason we are building the automatic goniometer with LCTF camera starting from summer of 2016 in Hokkaido University. The goniometer system is a mechanical instrument for changing the position of the light source and observation view, and operation of the system is synchronized with a software of LCTF imager on the computer. This system consists of four rotating axes. The view zenith has 92 cm radius of half arch, 115 cm long and 88 cm height of parallelepiped arch with a camera stand mount. It can fix the position of the camera from 0^o^ to 90^o^; the relative azimuth is the camera referenced from the light source on a horizontal plane, in a clockwise direction. Light source axis has an aluminium 75cm long arm which is also the distance between measurement target and illumination. The light source, relative azimuth, and rotating polarizer axes have motorized rotating stage which is the choice for many precision motion control applications and made in by JY Instrument Co., Ltd. Light zenith angle's stepper motor one step precision is 2 degrees. A stepper motor connected to worm gear with a ratio of 1:200 inside the rotating stages with the 2 degrees accuracy of stepper motors. It means the goniometers minimum movement step is 0.01 degree for illumination zenith and relative azimuth axes. For polarizer axis, we used 1:3 ratio of two circle gears with a 7.2-degree accuracy of the stepper motor. Electronics hardware of the goniometer is RAMPS 1.4 control board of open hardware 3D printer. Authors of this paper developed software of the system at the winter of 2016.

The goniometer operation is automatically changed position for illumination zenith from 0^o^ to 72^o^ with a step of 12^o^, relative azimuth from 10^o^ to 180^o^ degree with a step of 10^o^, and rotating linear polarizer 0^o^, 45^o^, 90^o^, 135^o^, respectively. Observation zenith angles are fixed manually at 0^o^, 12^o^, 24^o^, 36^o^, 42^o^, 48^o^, 54^o^, 60^o^, 66^o^, and 72^o^. LCTF camera can select every single wavelength band between 460 to 780 nm and take pictures on any arbitrary steps. We chose may influence and important 36 bands {460, 466, 472, 478: β- carotene tallest absorption peak, 485, 492, 500, 510, 518: β- carotene last absorption peak, 525, 535, 550, 560, 570, 585, 600, 615, 628, 636, 642: Second peak of chlorophyll b, 646, 650, 656, 662, 668, 674, 680, 687, 695, 703, 710, 725, 740, 750, 765, 780 nm}.

### 4.3 BRDF measurements

BRF is defined as the ratio between radiant flux, $d\Phi_{r}$ of the leaf and an ideal radiant flux, $d\Phi_{r}^{id}$ which would be reflected by an ideal (lossless) lambertion surface [7], [8], [9].

(2) $$BRF\left(\theta_{i},\phi_{i},\theta_{r},\phi_{i},\lambda \right)=\frac{d\Phi_{r}}{d\Phi_{r}^{id}}=\frac{dA\int_{\omega r}\int_{\omega i}f_{r}\left(\theta_{i},\Phi_{i},\theta_{r},\phi_{r}\lambda \right)L_{i}\left(\theta_{i},\phi_{i},\lambda \right)d\Omega_{r},d\Omega_{i}}{\frac{dA}{\pi }\int_{\omega r}\int_{\omega_{i}}E\left(\theta_{i},\phi_{i},\lambda \right)d\Omega_{r},d\Omega_{i}}$$

Eq. (2) where $E(\theta_{i},\;\phi_{i},\lambda )$ is incident radiance coming from the light source, and an ideal Lambertian surface reflects the same radiance all way direction. Notifications are explained in Table 1.

**Table 1:** Notations for BRF measurements.

Symbols	Quantity and unit	*Subscripts and superscripts*	Description
L	Radiance, [W m^−2^ sr^−1^ nm^-1^]	i	Incident or illumination
*E*	Irradiance, incident flux density; ≡ dΦ/dA [W m^−2^]	r	Reflected
*A*	Surface area [m^−2^]	id	Ideal (lossless)
Φ	Radiant flux [W]	p	Polarized
λ	Wavelength of radiation [nm]	w	Waxy cuticle
ρ	Reflectance; ≡ dΦ_r_/dΦ_i_	up	Unpolarized
R	Reflectance factor; ≡$d\Phi_{r}$ / $d\Phi_{r}^{id}$	diff	Diffuse
θ	Zenith angle, [rad]	spec	Specular

When those radiant fluxes are measured with the same device, the surface area *dA* of reflected flux must be same as ideal Lambertian surface area *dA*. The corresponding Eq. (2) BRF is π times higher than Bidirectional Reflectance Distribution Function (BRDF). Reflectance factor becomes a ratio of the reflected radiance of the target leaf to the reflected radiance of an ideal Lambertian surface at the identical view and illumination geometry. Azimuth view $\;\phi_{r}$ and azimuth incident $\;\phi_{i}$ angles are relative to the coordinate system; however, the two angles can be replaced by relative azimuth $\phi =\;\phi_{i}-\;\phi_{r}$ in a laboratory measurement. We added new parameter polarizer rotation angle α on the BRF measurement for relationship bewtween bidirectional reflectance and polarization. This additional variable doesn't change definition of relevant quantities, and number of measurements will increase at the one point.

(3) $$BRF\left(\theta_{i},\;\phi ,\;\theta_{r},\lambda ,\alpha \right)=\pi \cdot BRDF\left(\theta_{i},\;\phi ,\;\theta_{r},\lambda ,\alpha \right)=\frac{L_{r}\left(\theta_{i},\;\phi ,\;\theta_{r},\lambda ,\alpha \right)}{L_{r}^{id}\left(\theta_{i},\lambda \right)}$$

where $L_{r}^{id}$ is reflected radiance of ideal Lambertian (diffuse) surface and does not depend on relative azimuth, zenith view, and polarizer rotation angle. For an ideal Lambertian surface, high electron density of barium sulphate is selected, it is white crystalline powder which has a long tradition as a standard material for diffuse reflectance measurements.

Reflected radiance $L_{r}^{id}\;$can be written from the incident angle $\theta_{i}$ as [9]:

(4) $$L_{r}^{id}\left(\theta_{i},\lambda \right)=\frac{L_{r}^{Ba}\left(0,\lambda \right)\cdot cos\theta_{i}}{\rho^{Ba}}$$

where $L_{r}^{Ba}\left(0,\lambda \right)$ is spectral radiance of BaSO_4_ plate at illumination from the highest position ($\theta_{i}=0$) and $\rho^{Ba}$ is the reflectance of barium sulphate plate which was determined by laboratory measurement in lots of papers. Spectral radiance is based on the digital number of one pixel by Eq. (1), and one pixel's ground FOV is 0.15 × 0.15 mm for nadir view. If observation view zenith angle $\theta_{r}$ is going to increase (slope view), vertical side of ground FOV will be divided by $cos\theta_{r}$. When the viewable (visible) area decreases at the shallow angle, on the other hand, the area corresponding to a pixel increases so that radiance will be constant in this case. Radiances of at least 45 pixels are averaged from a visible area of the leaf for reducing the error of measurement and number of pixels depends on view zenith angle$\;\;\theta_{r}$.

The Directional Hemispheric Reflectance factor (DHRF) has an important role for measuring the optical property of the leaf, it is often measured by spectrophotometer equipped with integrating spheres [10]. In this analysis, DHRF is defined as:

(5) $$DHRF\left(\theta_{i},\lambda \right)=\int_{0}^{2\pi }\int_{0}^{\pi /2}BRDF\left(\theta_{i},\;\phi ,\;\theta_{r},\lambda \right)\;\times cos\theta_{r}sin\theta_{r}d\theta_{r}d\varphi$$

### 4.4 Polarization analysis

The polarization property of reflectance can be described by Stokes parameters (*I, Q, U*, and *V*). *V* describes the circularly polarized reflectance; therefore, this parameter usually can be neglected for light reflection on the leaf [11].

(6) $$I=\left(L_{0^{o}}+L_{45^{o}}+L_{90^{o}}+L_{135^{o}}\right)/2$$

(7) $$Q=L_{0^{o}}-L_{90^{o}}$$

(8) $$U=L_{45^{o}}-L_{135^{o}}$$

(9) $$DoLP=\sqrt{Q^{2}+U^{2}}/\;I=-Q/I$$

where $L_{0^{o}},L_{45^{o}},L_{90^{o}}$ and $L_{135^{o}}$ are obtained for 0°, 45°, 90°, and 135° linear polarizer directions those angles are relative to the vertical axis of camera lens plane, and the meaning of I is the total reflected radiance ($I=L_{r}^{total}$). The sum of polarized and unpolarized radiances are the total radiance ($L_{total}=L_{p}+L_{up}$) those two parts can be found below:

(10) $$L_{p}=\sqrt{Q^{2}+U^{2}}\;$$

(11) $$L_{up}=I-\sqrt{Q^{2}+U^{2}}\;$$

The polarized reflectance factor is able to express by the polarized reflected radiance with a barium sulphate reference:

(12) $$R_{p}\left(\theta_{i},\;\phi ,\;\theta_{r},\lambda \right)=\;\frac{L_{p}\left(\theta_{i},\;\phi ,\;\theta_{r},\lambda \right)}{L_{r}^{Ba}\left(\lambda \right)\cdot cos\theta_{i}}$$

(13) $$R_{up}\left(\theta_{i},\;\phi ,\;\theta_{r},\lambda \right)=\;\frac{L_{up}\left(\theta_{i},\;\phi ,\;\theta_{r},\lambda \right)}{L_{r}^{Ba}\left(\lambda \right)\cdot cos\theta_{i}}$$

The polarized reflectance can be written as

(14) $$BRF_{pol}=Q_{spec}\cdot BRF_{spec}+Q_{diff}\cdot BRF_{diff}$$

(15) $$BRF_{up}=BRF_{total}-BRF_{pol},$$

(16) $$Q_{spec}=\frac{R_{spec}^{⊥}-R_{spec}^{∥}}{R_{spec}^{⊥}+R_{spec}^{∥}},$$

(17) $$Q_{diff}=\frac{R_{diff}^{⊥}-R_{diff}^{∥}}{R_{diff}^{⊥}+R_{diff}^{∥}}\;.$$

For a polarization dependent function of the diffuse component ($Q_{diff}$), the minimum and the maximum values of reflectance factors correspond to parallel and perpendicular directions, respectively. $Q_{diff}$ does not depend on the relative azimuth angles, and is isotropic.

## Limitations

In this paper, only the leaves of the coffee plant are displayed. Due to the large amount of data generated during the measurement period, there is limited opportunity to display multiple data and different plant measurements.

## Ethics Statement

This work does not include human and animal experiments, as well as data in this article did not collected from social media.

## CRediT authorship contribution statement

**Begzsuren Tumendemberel:** Conceptualization, Methodology, Software, Data curation, Writing – original draft, Validation, Visualization. **Luvsanbat Khurelbaatar:** Writing – review & editing, Visualization. **Yukihiro Takahashi:** Investigation, Supervision. **Turtogtokh Tumenjargal:** Writing – review & editing. **Erdenebaatar Dashdondog:** Supervision, Writing – review & editing.

## Data Availability

Measurement of single Coffea canephora leaf spectro-polarimetric Bidirectional Reflectance Factor dataset and prediction model (Original data) (Mendeley Data).
